# Decoupling effect and driving factors of carbon footprint in megacity Wuhan, Central China

**DOI:** 10.1186/s13717-023-00435-y

**Published:** 2023-05-17

**Authors:** Gao Pan, Xinhang Li, Deng Pan, Wensheng Liu

**Affiliations:** 1 College of Life Science and Technology, Central South University of Forestry and Technology, Changsha, 410004 People’s Republic of China; 2 Central South Academy of Inventory and Planning of NFGA, Changsha, 410014 People’s Republic of China

**Keywords:** Carbon footprint, Economic development, Decoupling analysis, Partial least squares analysis, Megacity

## Abstract

**Background:**

China’s 35 largest cities, including Wuhan, are inhabited by approximately 18% of the Chinese population, and account for 40% energy consumption and greenhouse gas emissions. Wuhan is the only sub-provincial city in Central China and, as the eighth largest economy nationwide, has experienced a notable increase in energy consumption. However, major knowledge gaps exist in understanding the nexus of economic development and carbon footprint and their drivers in Wuhan.

**Methods:**

We studied Wuhan for the evolutionary characteristics of its carbon footprint (CF), the decoupling relationship between economic development and CF, and the essential drivers of CF. Based on the CF model, we quantified the dynamic trends of CF, carbon carrying capacity, carbon deficit, and carbon deficit pressure index from 2001 to 2020. We also adopted a decoupling model to clarify the coupled dynamics among total CF, its accounts, and economic development. We used the partial least squares method to analyze the influencing factors of Wuhan’s CF and determine the main drivers.

**Results:**

The CF of Wuhan increased from 36.01 million t CO_2_eq in 2001 to 70.07 million t CO_2_eq in 2020, a growth rate of 94.61%, which was much faster than that of the carbon carrying capacity. The energy consumption account (84.15%) far exceeded other accounts, and was mostly contributed by raw coal, coke, and crude oil. The carbon deficit pressure index fluctuated in the range of 8.44–6.74%, indicating that Wuhan was in the relief zone and the mild enhancement zone during 2001–2020. Around the same time, Wuhan was in a transition stage between weak and strong CF decoupling and economic growth. The main driving factor of CF growth was the urban per capita residential building area, while energy consumption per unit of GDP was responsible for the CF decline.

**Conclusions:**

Our research highlights the interaction of urban ecological and economic systems, and that Wuhan’s CF changes were mainly affected by four factors: city size, economic development, social consumption, and technological progress. The findings are of realistic significance in promoting low-carbon urban development and improving the city’s sustainability, and the related policies can offer an excellent benchmark for other cities with similar challenges.

**Supplementary Information:**

The online version contains supplementary material available at 10.1186/s13717-023-00435-y.

## Introduction

Greenhouse gas (GHG) emission over many years is one of the most important factors affecting climate change, and also is one of the crucial environmental concerns of humankind. These concerns have become more severe in China following economic development and population growth. Cities are centers of politics, economy, culture, population, and transportation (Lu et al. 2016). Over the past decades, urbanization in China, as a developing country, began later than in other places but has experienced dramatically rapid expansion, which has also induced the development of megacities (Fang et al. 2016). Since the reform and opening up, China’s urbanization rate has increased from 17.92% to 63.89% in 2020 (Cai et al. 2020). Meanwhile, urban land area in China has increased by more than eight times (from 7438 to 60,721 km^2^) between 1981 and 2020. Notably, China’s 35 largest cities, such as Wuhan, include approximately 18% of the population, and account for 40% of the energy consumption and greenhouse gas (GHG) emissions (Wang et al. 2018). Moreover, fulfilling the national commitment to reduce carbon emission intensity by approximately 65% relative to 2005 has been a great barrier. Therefore, it is crucial to analyze GHG emission trends and clarify the essential drivers, which also contribute to government scientific policies.

To study GHG emissions from megacities, one of the common international approaches is using the carbon footprint (CF). CF originated from the ecological footprint concept and later became an independent concept (East 2008). It is a comprehensive indicator to measure the CO_2_ or GHG emissions generated during production and the full lifecycle (Pan and Zhang 2021). More importantly, CF is a straightforward method that can reflect the influence of human activities on ecosystems and further reveal the interactions between people and ecosystems. Based on these benefits, CF has received increasing attention from researchers, who have mainly focused on the definitions and methodologies of CF (Lombardi et al. 2017), quantification of CF at different scales (Long et al. 2019), CF of various crop production from different areas (Zhang et al. 2017; Tian et al. 2021), analysis of CF drivers and decomposition models (Li et al. 2022a, b), and the nexus of CF and economic development (Mi et al. 2020). Previous studies have already demonstrated the usefulness of CF and offered a scientific basis for further research. However, there is a shortcoming: the key factors driving CF changes are difficult to determine. To remedy this deficiency, the application of CF is usually combined with other approaches, such as input–output analysis (Su et al. 2017), partial least squares (PLS) method (Huang 2019), structural decomposition analysis (Huang et al. 2019), and logarithmic mean Divisia index method (Chong et al. 2015; Zhen et al. 2017). Among them, the PLS method complements CF and overcomes its drawbacks (Yang et al. 2015).

As proposed by Wold and Albano in 1983, the PLS method has become a widely used tool in stoichiometry, industrial design, market analysis, economics, and biomedicine (Bayer et al. 2012). The method can cope with multicollinearity problems even when the number of variables is high, and it simultaneously integrates multiple regression, principal component analysis, and canonical correlation analysis into a model (Monecke and Leisch 2012). More importantly, it can decompose and screen data information in the system, extract the aggregate variables having the greatest impact on the dependent variable, and identify the information and noise in the modeling process (Yang et al. 2015); thus, it appears to be more appropriate in analyzing CF drivers than other approaches. As such, we use the PLS method to analyze the essential drivers of CF and clarify the nexus of carbon emission reduction and economic development. Decoupling analysis is an effective tool to explore the relationships among energy, environment, and economy (Grand 2016). Moreover, the decoupling model is often combined with decomposition methods, such as the PLS method. The PLS method can identify the important drivers for CF, and decoupling analysis allows us to understand the contribution of these factors to the evolution of the decoupling process in a given city. However, few studies have focused on the decoupling mechanism of economic development and CF. In this regard, several questions need to be answered. For example, the decoupling mechanism between CF and GDP year-by-year is unknown as are the essential decoupling drivers. These questions are pivotal for a city to establish energy-saving and carbon emission reduction policies, especially for a large city like a megacity.

To achieve a win–win situation for economic growth and environmental improvement, it is necessary to clarify the main factors driving the CF in Wuhan and the characteristics of various factors at different stages of economic development and propose carbon emission policies for these different stages. The objectives of this study were to: (1) quantitatively analyze the dynamic trends of CF, carbon carrying capacity (CC), and carbon deficit (CD) in Wuhan during 2001–2020; (2) identify the decoupling relationship between the total CF and its accounts and economic development in Wuhan; and (3) find out the main driving factors of CF changes in the region. This study offers an excellent benchmark for the government to evaluate the influence of their policies on carbon emission mitigation, and also helps to elaborate more suitable polices for achieving sustainable development of ecosystems and economic and social systems.

## Materials and methods

### Overview of Wuhan, China

**Fig. 1:**
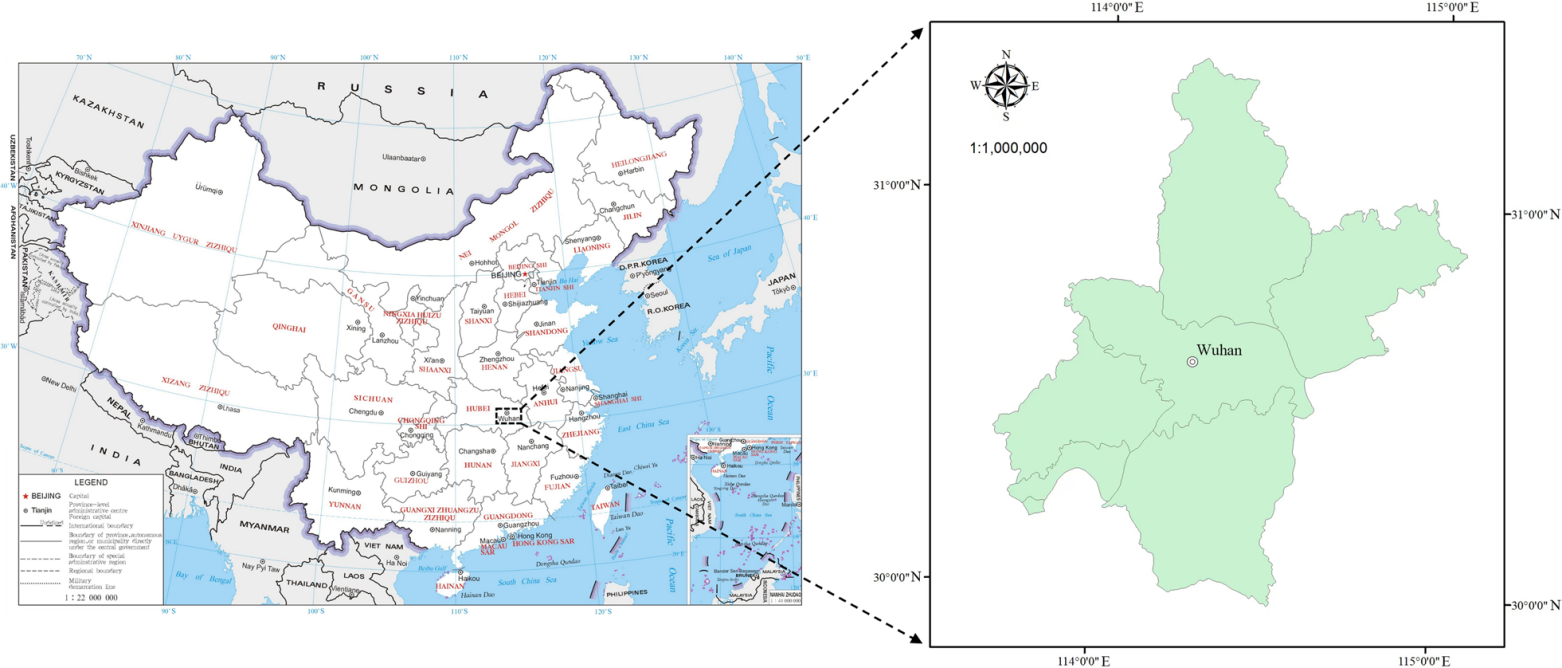
Location of Wuhan in China

Wuhan City, which lies between 29° 58′–31° 22′ N and 113° 41′–115° 05′ E, is located in central China (Fig. 1). Wuhan is the political, economic, and cultural center of Hubei Province and is also the only sub-provincial city in Central China. The city has jurisdiction over thirteen districts, with a total area of 8569.15 km^2^ and home to 13.65 million people. As the eighth largest economy nationwide, its GDP reached 1771.68 billion RMB in 2021 (Li et al. 2022a, b). The added value of its primary, secondary, and tertiary industries increased from 8.14 billion RMB, 53.33 billion RMB, and 59.22 billion RMB in 2000 to 40.22 billion RMB, 555.75 billion RMB, and 965.64 billion RMB in 2020. The composition of the three industries is 2.6:35.6:61.8 (Wuhan Statistics Bureau 2021). The rapid economic development of Wuhan induced an obvious increase in energy consumption. By 2020, the usage of coal (2075.47 × 10^4^ t) and natural gas (144,313.62 × 10^4^ m^3^) increased nearly two times and 210 times, respectively, compared to 2000.

### Carbon footprint model

In this study, we adopted a combination of the Intergovernmental Panel on Climate Change (IPCC) emission inventory and *Guidelines for the Preparation of Provincial Greenhouse Gas Inventories* to account for regional GHG emissions. We modified the carbon footprint model and constructed four accounts based on emission sources to analyze the CF of Wuhan, which including the energy consumption (CF_*e*_), industrial production process (CF_*p*_), polluting emission (CF_*w*_), and livestock accounts (CF_*l*_). The formula used was as follows:

1 $${\text{CF}} = {\text{CF}}_{e} + {\text{CF}}_{p} + {\text{CF}}_{w} + {\text{CF}}_{l}$$

#### Energy consumption account

Carbon emissions from fossil energy are mainly emitted as CO_2_, with only a small amount in the form of CH_4_ and N_2_O. Hence, our study only considered CO_2_ emissions in the energy consumption account. According to the IPCC guidelines 2006, Eqs. (2), (3), and (4) were formulated to calculate CF_*e*_ for fossil fuel combustion:

2 $${\text{CF}}_{e} = {\text{CF}}_{e1} + {\text{CF}}_{e2}$$

where CF_*e1*_ and CF_*e2*_ are the CF from the fixed and mobile source of the energy consumption account, respectively.

3 $${\text{CF}}_{e1} = \, \sum \, \left( {{\text{AC}}_{j} \times {\text{NCV}}_{j} \times {\text{EF}} \times {\text{COF}}_{j} \times \, 10^{ - 6} } \right) \, \times \, 44/12$$

where AC_*j*_ refers to the amount of the *j*th fuel (10^4^ t or 10^4^ m^3^); NCV_*j*_, EF_*j*_, and COF_*j*_ are shown in Additional file 1: Table S2; 10^–6^ indicates the unit conversion factor; and 44/12 refers to the ratio of CO_2_ emission and carbon chemical molecular weight:

4 $${\text{CF}}_{e2} = \, \sum \, \left( {{\text{VP}}_{i} \times {\text{VMT}}_{i} \times {\text{FE}}_{ig/d} \times {\text{EF}}_{g/d} } \right),$$

where VP_*i*_, VMT_*i*_, FE_*ig/d*_, and EF_*g/d*_ refer to the number, annual average mileage (km/vehicle), fuel economy (L/km), and carbon emission factor (t C/t) of the *i*th vehicle, respectively, and *g* and *d* represent gasoline and diesel.

#### Industrial production process account

Since 1985, China has become the largest global producer and consumer of cement (Shen et al. 2016). During industrial production, the largest fraction of carbon emissions is related to cement output, which has been calculated (Kajaste and Hurme 2016). However, fossil fuels from industrial production processes include CO_2_ emitted by the fuel used; thus, carbon emissions from the decomposition and transformation of raw materials are considered in this study. The emission factor of clinker is 0.538 t CO_2_/t, which can be retrieved from the *Guidelines for the Preparation of Provincial Greenhouse Gas Inventories*:

5 $${\text{CF}}_{p} = S_{y} \times D$$

where *S*_*y*_ refers to the output of Portland cement clinker, and *D* represents the emission factor of clinker.

#### Polluting emission account

Owing to the standardized calculation process and accurate results, we adopted the first-order attenuation method to calculate GHG emissions of solid waste, which has also been recommended by the *2006 IPCC Guidelines for National Greenhouse Gas Inventory*:

6 $${\text{DDOC}}_{mdT} = W_{T} \times {\text{DOC}} \times {\text{DOC}}_{f} \times {\text{MCF}}$$

where DDOC_*mdT*_ represents the decomposable carbon in *T*year landfill in an anaerobic environment, and *W*_*T*_ indicates the volume of industrial solid waste treated in *T*year (10^4^ t). DOC, DOC_*f*_, and MCF were considered using the method proposed by Qu and Yang (2011):

7 $${\text{CH}}_{4,\text{produce},T} = {\text{DDOC}}_{mdT} \times F_{a} \times \, 16/12$$

8 $${\text{CO}}_{2,\text{produce},T} = {\text{DDOC}}_{mdT} \times F_{b} \times \, 44/12$$

where *F*_*a*_ and *F*_*b*_ are the volume proportions of CH_4_ and CO_2_ in the total gas generated during landfill, respectively; 16/12 indicates the ratio of CH_4_ emissions to carbon chemical molecular weight; and 44/12 represents the ratio of CO_2_ emissions to carbon chemical molecular weight.

In this study, CF_*w1*_, CF_*w2*_, and CF_*w3*_ represent the emissions of solid waste, CH_4_, and N_2_O (10^4^ t), respectively. CF_*w1*_ can be calculated as the sum of CH_4*,*emission*,T*_ and CO_2,emission*,T*_:

9 $${\text{CO}}_{{2,{\text{emission}},T}} = \, \left(\sum {\text{CO}}_{{2,{\text{produce}},X,T}} - R_{T} \right) \, \times \, (1 - {\text{OX}}_{T} )$$

10 $${\text{CO}}_{{2,{\text{emission}},T}} = \, \left(\sum {\text{CO}}_{{2,{\text{produce}},X,T}} - R_{T} \right) \, \times \, (1 - {\text{OX}}_{T} )$$

where *R*_*T*_ refers to the recovery of CH_4_ and CO_2_, and OX_*T*_ is the oxidation factor:

11 $${\text{CF}}_{w2} = T \times B_{0} \times {\text{MCF}} - R$$

where *T* refers to the degradable organic matter (BOD) and can be obtained by multiplying the chemical oxygen demand (COD) removal by the average value of BOD/COD. *MCF* represents the correction factor of methane, *R* indicates the recovery of CH_4_:

12 $${\text{CF}}_{w3} = \, [(P \times P_{r} \times F_{{{\text{NPR}}}} \times F_{{\text{NON - CON}}} \times F_{{\text{IND - COM}}} ) - N_{S} ] \, \times {\text{EF}}_{E} \times \, 44/28 \, \times \, 10^{ - 7}$$

where *P* refers to the human population, *P*_*r*_ indicates the per capita consumption of protein, *F*_*NPR*_ represents the nitrogen mass in protein, *F*_NON–CON_ is the factor for non-consumed protein, *F*_IND–COM_ indicates the emission factor of protein, *N*_*S*_ represents the nitrogen disposed of sludge, and EF_*E*_ refers to the emission factor of N_2_O.

#### Livestock account

Agriculture provides 24% of the global GHG emissions and still growing each year (Bai et al. 2018). Approximately 32.56 × 10^9^ t of CO_2_eq GHG is derived from livestock and its by-products, accounting for 51% of global emissions. Therefore, the CF of livestock should be included in the calculation of Wuhan’s CF, which plays a pivotal role in evaluating the policies of carbon emission mitigation. In addition, CH_4_ accounts for about 44% of carbon emissions from livestock, so we focused on calculating CH_4_ emissions from livestock:

13 $$E_{{{\text{CH4,enteric,i}}}} = {\text{EF}}_{\text{CH4,enteric,i}} \times{\text{AP}}_{i} \times \, 10^{ - 7}$$

14 $${\text{CF}}_{l} = \, \sum E_{{{\text{CH4,enteric,i}}}}$$

where *E*_*CH4*_*,*_enteric*,i*_ and *EF*_*CH4*_*,*_enteric*,i*_ refer to the discharge amount and emissions factor of methane, respectively, and AP_*i*_ is the number of the *i*th animal.

### Carbon carrying capacity

CC is defined as the fixed CO_2_ absorbed by various vegetation types in a certain region every year (Mancini et al. 2016). Net ecosystem productivity (NEP) refers to the carbon sequestration capacity of vegetation. The main formula for calculating the *CC* of Wuhan is as follows (Fu et al. 2020):

15 $${\text{CC}} = C_{f} + C_{g} + C_{p}$$

16 $$C_{f} = M \times {\text{NEP}}_{f}$$

17 $$C_{g} = N \times {\text{NEP}}_{g}$$

18 $$C_{p} = \, 44/12 \, \times \lambda \times z \times \, \sum \, [P_{j} (1 - \omega_{j} )/O_{j} ]$$

where *M* refers to the area of forests (10^4^ hm^2^); *N* indicates the area of grasslands (10^4^ hm^2^), NEP_*f*_ and NEP_*g*_ are the carbon sequestration abilities of forests and grasslands, respectively; 44/12 represents the conversion coefficient of *C*; *λ* refers to the correction factor; *P*_*j*_ indicates the production of crops (10^4^ t);* z* represents the conversion coefficient; and *ω*_*j*_ and *O*_*j*_ are the moisture content and economic coefficient of crops, respectively.

### Carbon deficit and carbon deficit pressure index

The CD in a region is the residual between CF and CC:

19 $${\text{CD}} = {\text{CF}} - {\text{CC}}$$

If CD > 0, the carbon emissions exceed the carrying capacity and carbon surplus in the area, which further warms the climate. If CD = 0, the carbon budget is balanced. If CD < 0, the sum of the fixed CO_2_ can be absorbed by carbon sinks in the area, which is conducive to slowing the warming process.

According to Lu et al. (2013), the CD pressure index (CDI) can be calculated using Eq. (20):

20 $${\text{CDI}} = \, ({\text{CF}}_{t} /{\text{CC}}_{t} {-}{\text{CF}}_{t - 1} /{\text{CC}}_{t - 1} ) \times \left( {{\text{CF}}_{t - 1} /{\text{CC}}_{t - 1} } \right)^{ - 1}$$

The classification of CDI change is designed as in Table 1.

**Table 1:** Classification of the intensity of the change in CDI

Ecological pressure grade zone	Relief	Mild enhancement	Moderate enhancement	Severely enhancement	Extremely enhancement
CDI value range (%)	(− ∞, 0)	(0, 100]	(100, 200]	(200, 500]	(500, + ∞]

### Tapio’s decoupling model

The decoupling model is employed to explore the nexus of economic development and energy consumption (Pan and Zhang 2021). Currently, the most popular methods used for decoupling involve the OECD decoupling model and Tapio’s decoupling model (Grand 2016). However, the former has some disadvantages, such as measurement errors in decoupling, because factors are chosen randomly or do not fully reveal the decoupling effect. To remedy these defects, Tapio (2005) proposed a decoupling model, which specifies that decoupling states are categorized into eight sub-categories, depending on the values of α_*n*_ (CF and GDP). The standard and explanation of decoupling relationship are shown in Additional file 1: Table S1. The formula used was as follows:

21 $$\alpha_{n} = \, ({\text{CF}}_{n} - {\text{CF}}_{n - 1} )/{\text{CF}}_{n - 1} \times \, [({\text{GDP}}_{n} - {\text{GDP}}_{n - 1} )/{\text{GDP}}_{n - 1} ]^{ - 1}$$

where α_*n*_ refers to the decoupling elasticity index. The subscripts *n* and *n*–1 represent the year *n* and *n*–1.

### Partial least squares regression

PLS regression is an invaluable method when independent variables have strong collinearity (Jia et al. 2009). In the model, the CF of Wuhan was selected as the dependent variable *Y*, and independent variables included *X*_1_, *X*_2_, …, *X*_*p*_ for population, GDP, and other factors. Furthermore, the variable importance plot (VIP) value reflects the importance of *X*_1_, *X*_2_, …, *X*_*p*_ to *Y*. The higher the VIP, the greater the influence of the variable on the dependent variable.

In this study, we adopted Simca-P 11.5 (Umetrics, Ume, Sweden) to analyze the major drivers of Wuhan’s CF. In many cases, Kaya’s identity has also been widely used for determining CF drivers (Jia et al. 2009). To objectively reflect the important CF change factors, researchers have optimized the critical factors of Kaya’s identity. For instance, Jia et al. (2009) found that human population, GDP, and percentage of urban population were the key drivers of Henan’s CF. Huang et al. (2021) reported that population scale, economic development, technological level, and vegetation quality were the four frameworks driving CF change. For the crucial factors influencing Wuhan’s CF changes, we selected mainly from the following categories: city size, economic development, social consumption, and technological progress. The ten sub-categories are listed in Table 2.

**Table 2:** The driving factor indicators of carbon footprint in Wuhan

Primary indicator	Secondary indicators	Independent variable
City size	Urbanization rate	*X*_1_
	Resident population	*X*_2_
Economic development	GDP	*X*_3_
	Proportion of added value of the secondary industry	*X*_4_
	Total investment in fixed assets	*X*_5_
Social consumption	Total retail sales of consumer goods	*X*_6_
	Per capita disposable income of urban residents	*X*_7_
	Per capita annual net income of rural households	*X*_8_
	Urban per capita residential building area	*X*_9_
Technological progress	Energy consumption per unit of GDP	*X*_10_

### Data sources

First, for the energy consumption account, the consumptions of various fuels are obtained from the *2006 IPCC Guidelines for National Greenhouse Gas Inventory* and the *China Energy Statistical Yearbook* (see Additional file 1: Table S2). The *NCV*, *EF*, and *COF* of energy refer to the *China Energy Statistical Yearbook*. Data on civil vehicles are obtained from the *Wuhan Statistical Yearbook (2002–2021)*, and the *VMT*, *FE*, and *EF* of the vehicle are based on the results of He et al. (2005). For the industrial production process account, the output of “Portland Cement Clinker” is not available in *Wuhan Statistical Yearbook*, so it was replaced by the output of “Cement.”

Second, the sources of pollution emissions were divided into municipal solid waste, industrial solid waste, urban domestic sewage, and industrial wastewater discharge. Solid wastes in most cities were landfilled; thus, we only calculated GHG emissions from landfill treatment. During the treatment of solid waste, the emissions mainly comprised CO_2_ and CH_4_, with little O_2_, N_2_, H_2_S, and other GHG. Therefore, for the pollution emission account, we only calculated the emissions of CO_2_ and CH_4_. Based on the given data availability, the amount of “Municipal Solid Waste” was replaced with “Treatment Capacity of Living Garbage.” When calculating the GHG emissions from industrial solid waste, the “Volume of Industrial Solid Wastes Treated” was obtained from the *Wuhan Municipal Solid Waste Pollution Prevention and Control Information Announcement*. Furthermore, CH_4_ and N_2_O have 21 and 310 folds the warming potential of CO_2_, respectively. The values of DOC, DOC_*f*_, MCF, *F*, and OX were 0.14, 0.5, 1, 0.5, and 0, respectively, in the disposal of municipal solid waste, whereas they were 0.15, 0.5, 1, 0.5, and 0, respectively, in the disposal of industrial solid waste (Qu and Yang 2011). When calculating CH_4_ and N_2_O emissions from wastewater treatment, the values of *B*_*0*_, MCF, *R*, *F*_NPR_, *F*_NON–CON_, *F*_IND–COM_, *N*_*S*_, and EF_*E*_ were 0.6, 0.165, 0, 0.16, 1.1, 1.25, 0, and 0.005, respectively, based on the research by Zhang et al. (2014). For the livestock account, based on the main livestock types in Wuhan, methane emissions from intestinal fermentation and feces were calculated. The CH_4_ emission factors were obtained from the *2006 IPCC Guidelines for National Greenhouse Gas Inventory* and the *Guidelines for the Preparation of Provincial Greenhouse Gas Inventories* (see Additional file 1: Tables S3 and S4).

Third, land type was used to calculate the CC, including forestland, grassland, and cropland. According to Yang and Meng (2019), the values of NEP_*f*_, NEP_*g*_, *λ*, and *z* were 13.97, 3.48, 0.07, and 0.5, respectively. When calculating CC, data on areas of forestland and grassland were obtained from the *Report on the State of Greening in Wuhan* (Wuhan Municipal Bureau of Landscape and Forestry) and the *General Land Use Planning in Wuhan (2006–2020)*. Using these data may not avoid discordance with the actual CC; however, it is undeniable that the authority, availability, and completeness of data have been comprehensively evaluated. In addition, the selection of *ω*_*j*_ and *O*_*j*_ according to Yan et al. (2018) with the values is listed in the Appendix (Additional file 1: Table S5).

## Results

### Carbon footprint, carbon carrying capacity, and carbon deficit analysis

The CF increased by 94.61% changing from 36.01 to 70.07 million t CO_2_eq during 2001–2020 (Additional file 1: Fig. S1). From 2001 to 2006, CF exhibited rapid growth; after 2006, CF entered a period of fluctuating growth and exhibited a downward trend from 2020 onward. The CC of Wuhan fluctuated slightly between 2001 and 2007, and increased from 2.31 to 2.58 million t CO_2_eq. From 2008 to 2014, the CC decreased overall and remained essentially stable; after 2016, it reached 2.95 million t CO_2_eq, and then decreased gradually.

**Fig. 2:**
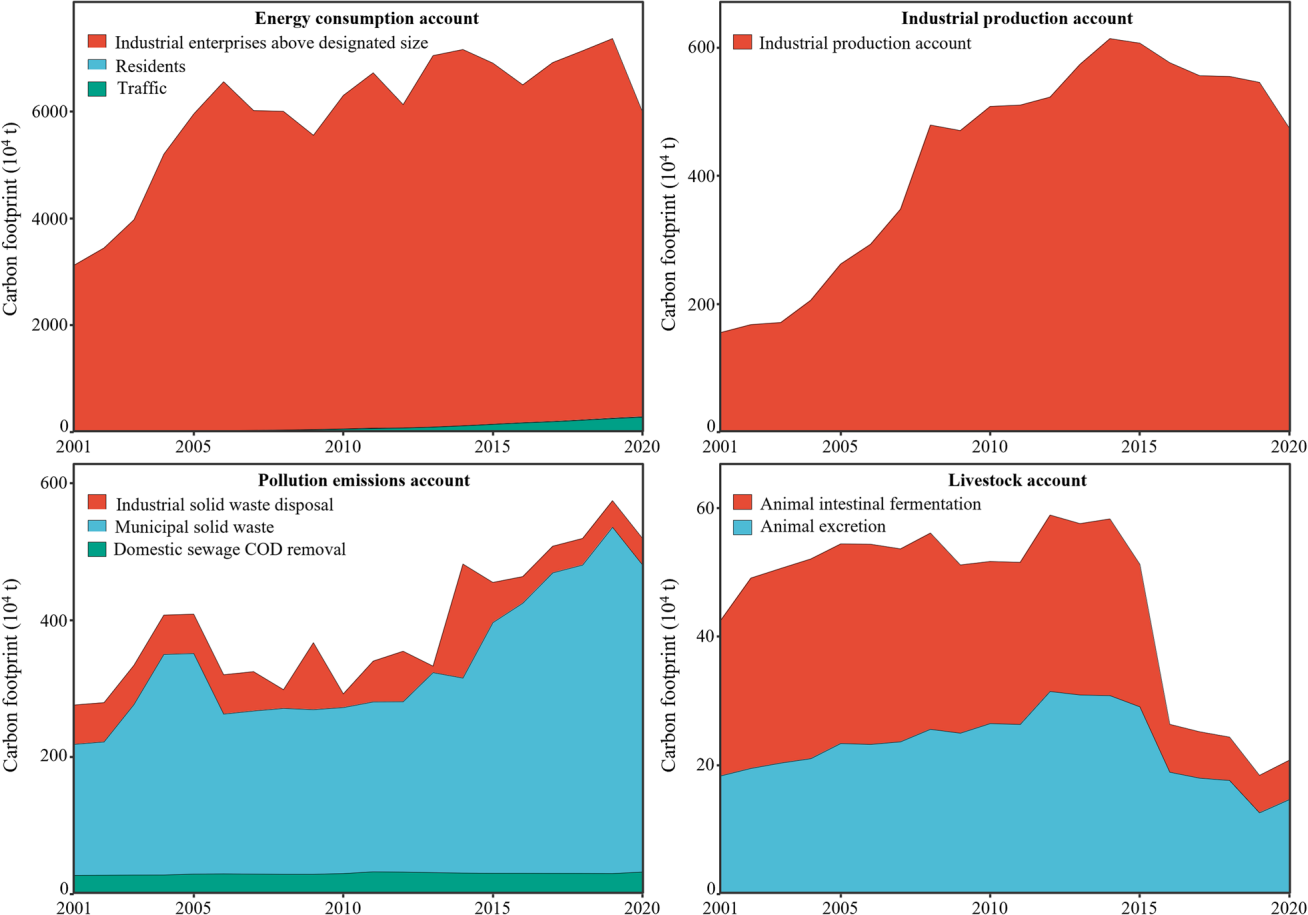
Energy consumption account, industrial production process account, pollution emission account, and livestock account of carbon footprint in Wuhan over 2001–2020

In the overall CF account, the energy consumption account occupied a dominant position, which showed an overall fluctuating upward trend (Fig. 2). The industrial production process account contributed the second largest share, an increase of 294.70%, and an average annual increase of 6.04%. The livestock account tended to be relatively stable before 2016 and then continuously decreased by 48.55% compared with 2015. The pollution emissions account showed erratic changes with alternate dips and rises, which fluctuated from 2.76 to 5.19 million t CO_2_eq with a growth rate of 88.09%.

From the perspective of energy consumption, industry had the greatest consumption, while transportation and residents accounted for a relatively small proportion (Fig. 2). Specifically, raw coal, coke, and crude oil contributed 96.06–99.47% to energy consumption, which dominated the CF of the industry. Municipal solid waste was the main contributor to the pollution emissions account and showed a fluctuating rising trend, followed by industrial solid waste and domestic sewage. Furthermore, the livestock account was dominated by methane, which was based on animal intestinal fermentation and feces. The CD of Wuhan exhibited an overall growth trend, from 33.70 million t CO_2_eq in 2001 to 67.85 million t CO_2_eq in 2020; the cumulative increase was 101.34%, with an average annual increase of 3.75% (Additional file 1: Fig. S1). Correspondingly, the growth in CF far exceeded that in CC. During the study period, the change in Wuhan’s CDI fluctuated in the range [8.44%, 6.74%] (Table 3).

**Table 3:** The change of carbon deficit pressure index in Wuhan from 2001 to 2020

Year	2001	2002	2003	2004	2005	2006	2007	2008	2009	2010	2011	2012	2013	2014	2015	2016	2017	2018	2019	2020
CDI	–	8.44%	15.22%	26.24%	34.75%	− 15.66%	− 6.62%	30.82%	− 9.68%	7.30%	7.40%	− 8.98%	12.36%	3.49%	− 27.10%	− 6.45%	7.89%	3.19%	3.42%	6.74%

### Decoupling analysis

During the study period, Wuhan experienced four decoupling states of CF and GDP (Table 4). Correspondingly, weak decoupling, strong decoupling, and expansion negative decoupling appeared nine (52.94%), five (29.41%), and two (11.76%), respectively, while decay decoupling appeared only once. The nexus of the energy consumption account and GDP was consistent with the relationship between CF and GDP during all periods, except 2007–2008 and 2009–2010. From 2001 to 2020, weak decoupling, strong decoupling, expansion negative decoupling, and decay decoupling appeared eight, six, three, and one time, respectively, between the industrial production process account and GDP. The relationship between the pollution emission account and GDP experienced weak decoupling, strong decoupling, expansion negative decoupling, and decay decoupling during 2001–2020. The frequencies of occurrences were 42.11%, 26.32%, 26.32%, and 5.26%, respectively. Regarding the relationship between livestock account and GDP, strong decoupling and weak decoupling appeared ten (55.55%) and six (33.33%), respectively, while strong negative decoupling and expansion negative decoupling appeared only once.

**Table 4:** Decoupling effect of carbon footprint and economic growth in Wuhan from 2001 to 2020

Year	Total carbon footprint		Energy consumption account		Industrial production process account		Pollution emission account		Livestock account	
	α_*n*_	State	α_*n*_	State	α_*n*_	State	α_*n*_	State	α_*n*_	State
2001–2002	0.97	Expansion connection	1.04	Expansion connection	0.79	Weak decoupling	0.13	Weak decoupling	1.54	Expansion negative decoupling
2002–2003	1.41	Expansion negative decoupling	1.45	Expansion negative decoupling	0.19	Weak decoupling	1.85	Expansion negative decoupling	0.29	Weak decoupling
2003–2004	1.84	Expansion negative decoupling	1.92	Expansion negative decoupling	1.27	Expansion negative decoupling	1.37	Expansion negative decoupling	0.18	Weak decoupling
2004–2005	0.68	Weak decoupling	0.71	Weak decoupling	1.36	Expansion negative decoupling	0.02	Weak decoupling	0.22	Weak decoupling
2005–2006	0.45	Weak decoupling	0.56	Weak decoupling	0.65	Weak decoupling	− 1.2	Strong decoupling	− 0.01	Strong decoupling
2006–2007	− 0.34	Strong decoupling	− 0.43	Strong decoupling	0.96	Expansion connection	0.07	Weak decoupling	− 0.07	Strong decoupling
2007–2008	0.05	Weak decoupling	− 0.01	Strong decoupling	1.37	Expansion negative decoupling	− 0.29	Strong decoupling	0.17	Weak decoupling
2008–2009	− 0.34	Strong decoupling	− 0.45	Strong decoupling	− 0.11	Strong decoupling	1.38	Expansion negative decoupling	− 0.53	Strong decoupling
2009–2010	0.73	Weak decoupling	0.89	Expansion connection	0.53	Weak decoupling	− 1.35	Strong decoupling	0.07	Weak decoupling
2010–2011	0.32	Weak decoupling	0.33	Weak decoupling	0.02	Weak decoupling	0.79	Weak decoupling	− 0.01	Strong decoupling
2011–2012	− 0.41	Strong decoupling	− 0.5	Strong decoupling	0.14	Weak decoupling	0.24	Weak decoupling	0.81	Expansion connection
2012–2013	1.04	Expansion connection	1.16	Expansion connection	0.76	Weak decoupling	− 0.48	Strong decoupling	− 0.18	Strong decoupling
2013–2014	0.26	Weak decoupling	0.11	Weak decoupling	0.48	Weak decoupling	3.06	Expansion negative decoupling	0.09	Weak decoupling
2014–2015	− 0.68	Strong decoupling	− 0.68	Strong decoupling	− 0.22	Strong decoupling	− 1.07	Strong decoupling	− 2.33	Strong decoupling
2015–2016	− 0.61	Strong decoupling	− 0.63	Strong decoupling	− 0.54	Strong decoupling	0.2	Weak decoupling	− 5.21	Strong decoupling
2016–2017	0.43	Weak decoupling	0.47	Weak decoupling	− 0.26	Strong decoupling	0.71	Weak decoupling	− 0.33	Strong decoupling
2017–2018	0.2	Weak decoupling	0.23	Weak decoupling	− 0.01	Strong decoupling	0.16	Weak decoupling	− 0.23	Strong decoupling
2018–2019	0.37	Weak decoupling	0.37	Weak decoupling	− 0.19	Strong decoupling	1.22	Expansion negative decoupling	− 2.81	Strong decoupling
2019–2020	4.7	Decay decoupling	4.97	Decay decoupling	3.5	Decay decoupling	2.56	Decay decoupling	− 3.43	Strong negative decoupling

### Driving factors analysis

In the first PLS component extraction, the cross-connect effectiveness Q_1_^2^ was 0.515 > 1–0.95^2^ = 0.0975. When the second component was extracted, the cross-connect effectiveness Q_2_^2^ was 0.466 > 0.0975. Therefore, the system extracted the first and second components, because the third component was − 0.0862. The explanation capacities of models *X* and *Y* were 0.982 and 0.823, Q^2^(cum) = 0.741 > 0.5, indicating that the regression model had a higher accuracy and stronger reliability.

In this study, samples were distributed in an ellipse based on the analysis principle of a singular point, suggesting that samples satisfied the requirements of the regression model and the result was reliable. The final standardized PLS regression equation is

22 $$Y \, = \, 4.872 \, + 0.161X_{1} + \, 0.067X_{2} + \, 0.043X_{3} + \, 0.452X_{4} + \, 0.141X_{5} + \, 0.101X_{6} + \, 0.059X_{7} + \, 0.029X_{8} + \, 0.534X_{9} - \, 0.059X_{10}$$

VIP is generally applied to determine the statistical importance of independent variable *X* on dependent variable *Y*. It is widely accepted that VIP values greater than 0.85 indicate that the dependent variable is “important.” The VIP values of the independent variables both exceeded 0.85, and they may be significant driving factors of Wuhan’s CF (Additional file 1: Fig. S2). The order of the driving factors was as follows (from high to low): *X*_9_ > *X*_4_ > *X*_1_ > *X*_5_ > *X*_6_ > *X*_7_ > *X*_3_ > *X*_10_ > *X*_8_ > *X*_2_.

## Discussion

### Carbon footprint, carbon carrying capacity, and carbon deficit of Wuhan

Overall, the change in CF in Wuhan primarily comprised two parts: initial high growth and then fluctuating increase. Wuhan is an important industrial base in China and the largest economic center of Central China and the middle reaches of the Yangtze River. The rapid economic development noticeably promoted Wuhan’s CF. Among the accounts, industrial enterprises above a designated size constituted a substantial share of the energy consumption account and contributed the most to the growth of Wuhan’s CF. Similarly, Li et al. (2017) reported that the energy consumption account made the largest contribution to Xi’an’s CF. In all the study years, raw coal was responsible for most of the CF, followed by coke, crude oil, and diesel oil, which is similar to the result of Huang et al. (2021). Municipal solid waste was the dominant source contributing to the CF growth in the pollution emissions account, whereas other sources, including industrial solid waste and domestic sewage, occupied a small percentage (Fig. 2). “Waste siege” occurs in many Chinese cities, including Wuhan, due to the growing urban population and the rapid economic growth (Chen et al. 2019). In addition, the cause of the decrease in the livestock account may be due to the Delineation and Implementation Plan of Prohibited, Restricted, and Suitable Breeding Areas for Livestock and Poultry in Wuhan, which clearly proposes establishing prohibited and restricted areas after 2016 and leads to the reduction of livestock.

CD is the most common parameter used for understanding the carbon sink potential in a city and evaluating the eco-safety level of a region (Pan and Zhang 2021). In this study, CD presented a rapid growth trend and was greater than 0 during 2001–2020. This trend indicates that the CO_2_ emissions of Wuhan exceeded its carbon sink capacity. Wuhan is in a carbon surplus state, primarily because its economy and society are developing quickly and continuously, resulting in increasing energy demand and intensity. In addition to the CD, the CDI is also crucial for evaluating the influence of anthropogenic activities on regional ecosystems over a period of time (Cao et al. 2017). By the intensity of the CDI change, the area can be divided into relief CDI ≤ 0, mild enhancement 0 < CDI ≤ 100, moderate enhancement 100 < CDI ≤ 200, severe enhancement 200 < CDI ≤ 500, and extreme enhancement zones CDI > 500 (Lu et al. 2013). According to this standard, Wuhan was mainly in the relief and mild enhancement zones during 2001–2020. This result once again showed that Wuhan was in a carbon surplus state, even though the CDI value decreased slightly in certain years. Consequently, it is necessary to enhance the environmental quality of Wuhan based on the intensity of CDI change.

### Relationship between the CF and economic growth in Wuhan

Decoupling analysis has been broadly applied to various fields, which can accurately determine the nexus of economic growth and CO_2_ emission in an area (Li et al. 2019). During the entire study period, Wuhan experienced different decoupling states. The relationship between CF and the GDP of Wuhan is unstable, with weak decoupling and strong decoupling occurring for much of the time. In 2001, influenced by the implementation of Wuhan’s “10th Five-Year Plan,” the growth in GDP and CF increased with the overall startup of various projects, and an expansion connection appeared. During 2002–2004, Wuhan’s economy slowed; however, its CF continued to grow. Consequently, the relationship between them changed to expansion negative decoupling. Meanwhile, China was approved for entry into the WTO, which may have resulted in international and domestic competition in the retail industry. From 2004 to 2019, Wuhan experienced weak decoupling, strong decoupling, and expansion connection, and then returned to weak decoupling (Table 4). During this period, Wuhan began to pay attention to energy-saving and emissions decrease, and was approved as the “resource-conserving and environment-friendly society” comprehensive reform experimental region in 2007. In addition, the country set Wuhan as one of its pilot cities for a national low-carbon city in 2012 and proposed that Wuhan should optimize its energy structure and promote low-carbon development of energy, industry, and construction. Furthermore, the “13th Five-Year Plan” and carbon peak action plan were implemented in Wuhan, which required implementation of a low-carbon circular economy. Such efforts have decelerated the growth in carbon emissions, while GDP has experienced a relatively rapid increase. In 2020, both GDP and the CF of energy consumption, industrial production processes, and pollution emissions decreased simultaneously; thus, the nexus among them presented decay decoupling. Notably, 2020 is a crucial time; the COVID-19 outbreak substantially affects all aspects of human productivity and life, and catastrophically influences China and the world economy.

Overall, the nexus of the energy consumption account and GDP is nearly identical to that of the relationship between CF and economic development. The relationship between livestock accounts and GDP is favorable, presenting weak decoupling and strong decoupling during most periods. Weak decoupling and strong decoupling occurred frequently within the relationship between the industrial production process account and GDP. The relationship between the pollution emission account and GDP mainly presented weak decoupling, strong decoupling, and expansion negative decoupling (Table 4). Under the background of rapid growth in the urban population and economy, the amount of municipal solid waste is continuously augmenting, which contributes to the pollution emission account growth. Moreover, due to the “11th Five-Year Plan” and the action of industrial energy conservation and emission reduction were carried out, and Wuhan has achieved good results in industrial structure adjustment, energy saving, and emission reduction. For this reason, the nexus of the industrial production process account and GDP tends to be favorable after 2008.

### Driving factors of the carbon footprint in Wuhan

Regarding the social system, the urban per capita residential building area ranked first among the influential factors, and the VIP value was up to 1.44. The government adopted a range of measures to increase the urban per capita residential building area, such as the “11th Five-Year Plan” for urban spatial layout and the “12th Five-Year Plan” for housing development. Nevertheless, the construction industry involves electricity, cement, and steel with enormous carbon emissions. Therefore, Wuhan should take measures towards a low-carbon economy and energy conservation, comprehensively promote green buildings, and continuously advance green, low-carbon, and sustainable urban development. The disposable income of urban residents is far above that of rural households. By 2020, the per capita disposable income for urban residents reached RMB 50,362 and for rural households reached RMB 31,150. This might be due to the different consumption habits of urban and rural residents. High-income people generate more carbon emissions due to diversified consumption patterns and higher consumption levels (Huang et al. 2022). Consequently, the contribution of urban consumption to CF far exceeded that of rural consumption.

Regarding economic development, the proportion of added value of the secondary industry and total investment in fixed assets as contributors to CF changes separately ranked second and fourth. It can be considered that the application of indicators from economic development is essential for explaining the change in CF in Wuhan. According to Hong et al. (2011), economic development and expansion are the predominant drivers of energy consumption growth in megacities, especially in secondary industries. From 2001 to 2020, the added value of Wuhan’s secondary industry increased from 59.50 billion to 555.75 billion Chinese Yuan and formed an industrial energy system dominated by coal and oil. During this period, the total investment in fixed assets increased to 843.13 billion Chinese Yuan, an increase of nearly 16.6 times. Total investment in fixed assets has promoted rapid economic growth, but it overwhelmingly depended upon fossil energy with high carbon emissions. The result of Lin (2016) is similar to our finding. In addition, Wuhan should optimize its industrial structure, transform the field of fixed assets investment, and push the economy towards high-quality development.

Regarding city size, the urbanization rate was second among all driving factors. Beginning in 2001, the urbanization rate in Wuhan increased from 59.20% to 74.68% by 2020, which was above the national average (63.89%) by 2020. Enormous amounts of arable and forest lands have been transformed to construction land under rapid urbanization (Zhen et al. 2017). In house-building and municipal infrastructure construction, the massive use of steel bars, cement, and vehicles has caused a surge in CO_2_ emissions. However, many rural laborers entering cities have improved the consumption level and demand for energy, electricity, and transportation, which are major industries that generate large quantities of carbon dioxide. Hao et al. (2016) promoted the theory of “multiple planning integration” urban areas, through encouraging people to utilize public service facilities, which may reduce carbon emissions, thereby solving the contradiction between resource demand and environmental protection and, consequently, improving the living environment and quality of life. The resident population is another important factor contributing to Wuhan’s CF. From 2001 to 2020, the resident population in Wuhan increased by 52.96%. The continuous population growth contributed to a greater resource need, causing a growth in resource consumption and carbon emissions from daily life.

Technological improvements have inhibited CF growth. Advances in technology can upgrade production and improve technology, which influences productivity. However, advances in technology can propel the application of energy conservation techniques, which influences carbon intensity. In this study, CF growth was inhibited by Wuhan’s energy consumption per unit of GDP. This is similar to the observations of Su et al. (2018), who reported that progress in both production and energy conservation technologies has constrained CF growth. Consequently, Wuhan should place greater emphasis on investment and innovation in technology, strengthen policy support efforts for technology research and application, and achieve sustainable development goals.

## Conclusions and policy recommendations

### Conclusions

Urban CF and economic development interact. To move toward sustainable economic and social development, it is crucial to understand the nexus between CF and economic development. From 2001 to 2020, Wuhan was in a carbon surplus state and under enormous pressure to reduce carbon emissions due to its CF being much larger than its CC. Among these accounts, the energy consumption account dominates Wuhan’s CF, and the energy consumption structure is mainly comprised of raw coal, coke, and crude oil. Furthermore, we found that Wuhan was mainly in the ecological stress relief zone and mild enhancement zone during 2001–2020. We further analyzed the decoupling relationships between CF and economic development. This confirmed that a transition stage exists in Wuhan between weak decoupling and strong decoupling. Our research identifies the important factors influencing Wuhan’s CF. Notably, the urban per capita residential building area is crucial in promoting Wuhan’s CF, while technological progress inhibits CF’s growth. Such findings provide valuable policy insights for low-carbon urban development and for continuous improvement of a city’s sustainability.

### Policy recommendations

First, Wuhan should rationally adjust its economic structure through gradual cessation of high energy consumption and high emission projects, actively developing knowledge and technology-intensive industries. As one of the three intelligence-intensive areas and comprehensive transportation hubs in China, Wuhan could fully exploit the advantages of high-tech and logistics industries. Moreover, the city can also support the development of the digital economy, culture, and tourism.

Second, Wuhan should optimize its energy structure by shifting from fossil fuels to clean and renewable energy sources. This study showed that Wuhan has gradually diminished its consumption of various fossil fuels in recent years. Solar, wind, tidal, and geothermal energy should be energetically popularized and applied. Wuhan will further develop geothermal energy in the 14th Five-Year Plan period because of its abundant resources, cleanliness, and stability. Preferable policies should be implemented to support citizens’ use of renewable energy.

Finally, capacity-building efforts must be strengthened to enhance public awareness. Improving public awareness is critical for cities to achieve sustainable development. Positive and beneficial capacity-building efforts involve TV, radio, internet, magazines, and billboards. The city government should provide an additional financial budget to support these activities.

## Supplementary Information

**Additional file 1: Table S1.** Decoupling state and explanation of the carbon footprint and economic growth. **Table S2.** Carbon emission coefficients for various types of fuels. **Table S3**. Animal intestinal fermentation CH_4_ emission factor. **Table S4.** Manure management CH_4_ emission factor. **Fig. S1.** Changes in CF, CC, and CD in Wuhan from 2001 to 2020. **Fig. S2.** VIP plot.

## Data Availability

The data sets used and/or analyzed during the current study are available from the corresponding author on reasonable request.

## Abbreviations

CF: Carbon footprint
GHG: Greenhouse gas
GDP: Gross domestic product
PLS: Partial least squares
CC: Carbon carrying capacity
CD: Carbon deficit
VIP: Variable important in projection
NCV: Average low calorific value
EF: Carbon content per unit calorific value
COF: Carbon oxidation rate
VMT: Annual average mileage
FE: Fuel economy
FOD: First-order attenuation method
DOC: Degradable organic carbon
*R*_T_: Amount of CH_4_ and CO_2_ recovered
OX: Oxidation factor
BOD: Degradable organic matter
MCF: Methane correction factor
CDI: Carbon deficit pressure index
