# Supplementary material for: Decoupling effect and driving factors of carbon footprint in megacity Wuhan, Central China
Source: Ecol Process. 2023 May 17;12(1):23. doi: 10.1186/s13717-023-00435-y (PMC10189220; doi:10.1186/s13717-023-00435-y)
Supplement: Supplementary file 1 — Additional file 1: Table S1. Decoupling state and explanation of the carbon footprint and economic growth. Table S2. Carbon emission coefficients for various types of fuels. Table S3. Animal intestinal fermentation CH4 emission factor. Table S4. Manure management CH4 emission factor. Fig. S1. Changes in CF, CC, and CD in Wuhan from 2001 to 2020. Fig. S2. VIP plot. [file 13717_2023_435_MOESM1_ESM.docx]

Table S1 Decoupling state and explanation of the carbon footprint and economic growth.

| Decoupling state | | δCF | δGDP | α_n_ | Explanation |
| --- | --- | --- | --- | --- | --- |
| Decoupling | Strong decoupling | <0 | >0 | α_n_<0 | CF declines, and GDP still shows a growing trend. |
|  | Weak decoupling | >0 | <0 | 0<α_n_<e_1_ | Both CF and GDP are growing, and the GDP growth rate is greater than the CF growth rate. |
|  | Decay decoupling | <0 | <0 | α_n_>e_2_ | Both CF and GDP are decreasing, and the rate of GDP decline is less than the rate of CF decline. |
| Negative Decoupling | Weak negative Decoupling | <0 | <0 | 0<α_n_<e_1_ | Both CF and GDP are decreasing, and the rate of GDP decline is greater than the rate of CF decline. |
|  | Strong negative Decoupling | >0 | <0 | α_n_<0 | CF is growing, and GDP is decreasing. |
|  | Expansion negative Decoupling | >0 | >0 | α_n_>e_2_ | Both CF and GDP are growing, and the GDP growth rate is less than CF growth rate. |
| Connection | Decay connection | <0 | <0 | e_1_<α_n_<e_2_ | CF and GDP remain in a relatively synchronous decline. |
|  | Expansion connection | >0 | >0 | e_1_<α_n_<e_2_ | CF and GDP maintain a relatively simultaneous growth trend. |

Note: There is no case of a constant carbon footprint and GDP, and thus, the case of δCF=0 and δGDP=0 is not considered.


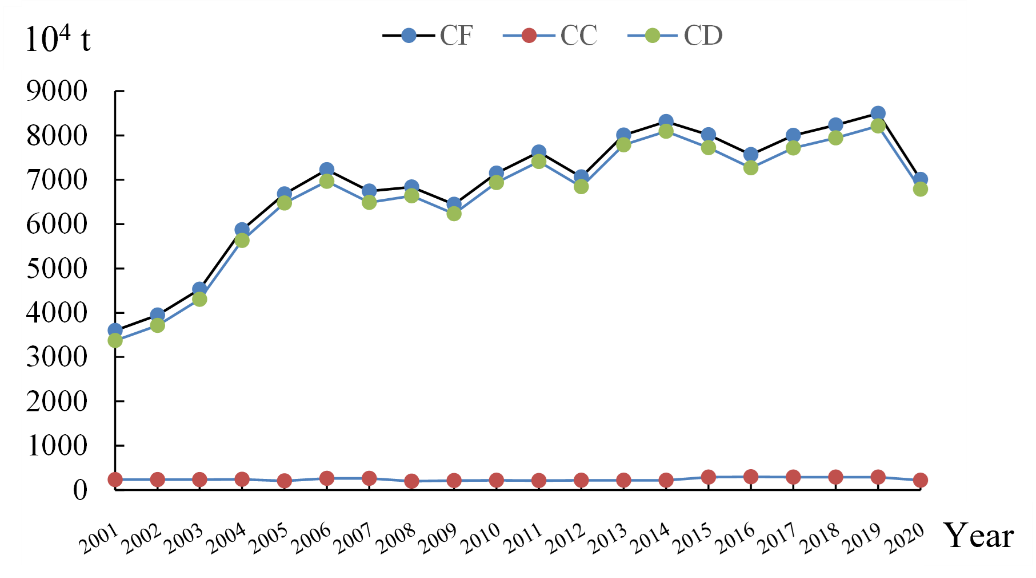


Fig. S1 Changes in the CF, CC, and CD in Wuhan from 2001 to 2020.


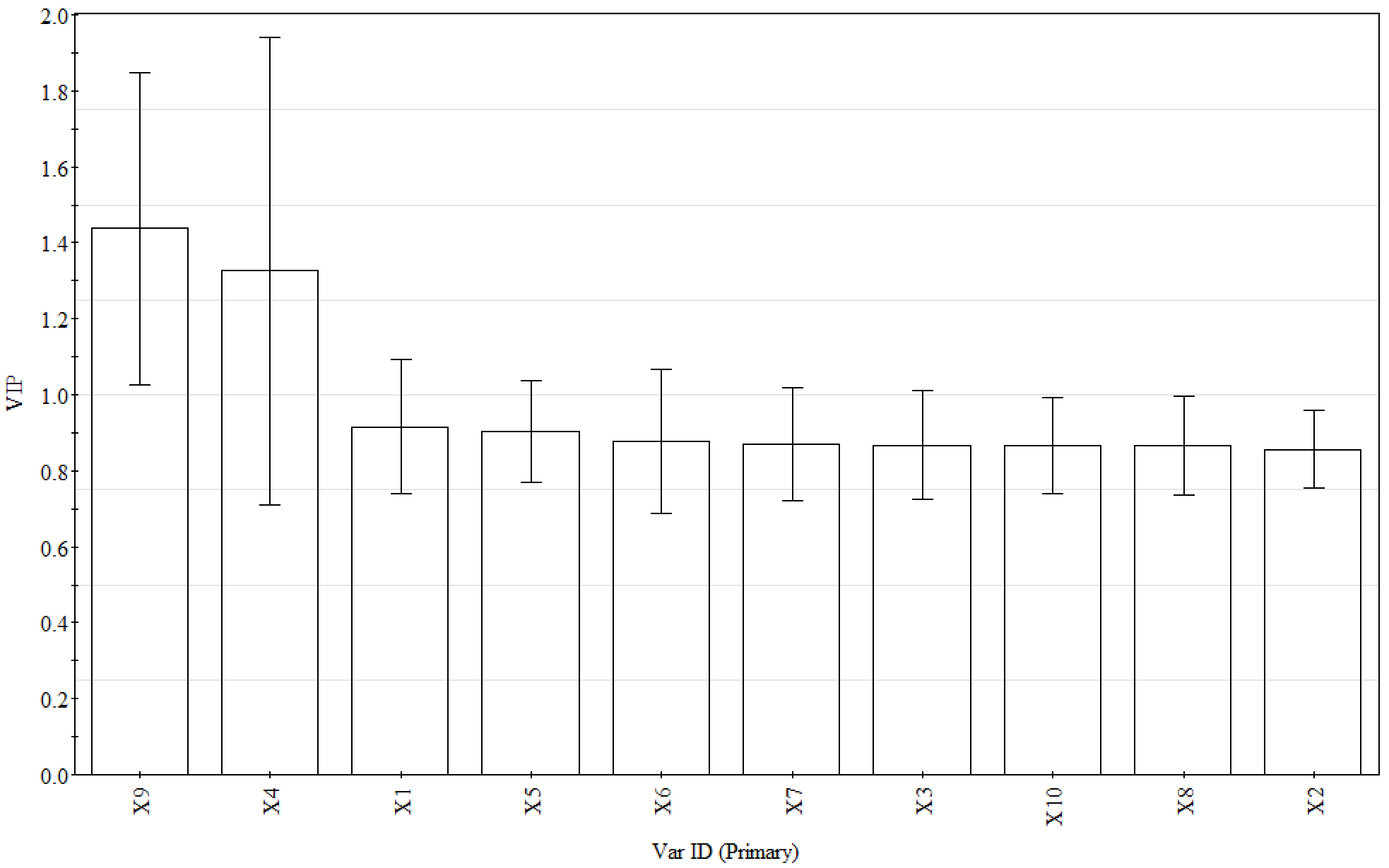


Fig. S2 The VIP plot.

Table S2 Carbon emission coefficient for various types of fuel.

| Fuel variety | Low calorific value (KJ/kg) | Carbon content (kg C/GJ) | Oxidation rate (%) | Carbon emission coefficient (t C/t) |
| --- | --- | --- | --- | --- |
| Raw coal | 20908 | 26 | 1 | 0.5394 |
| Coke | 28435 | 29 | 1 | 0.8303 |
| Crude | 41816 | 20 | 1 | 0.8363 |
| Gasoline | 43070 | 19 | 1 | 0.8140 |
| Diesel | 42652 | 20 | 1 | 0.8616 |
| Fuel oil | 41816 | 21 | 1 | 0.8823 |
| Natural gas | 38931 (KJ/m^3^) | 15 | 1 | 0.4478 |
| Kerosene | 43070 | 20 | 1 | 0.8399 |

Table S3 Animal intestinal fermentation CH_4_ emission factor

Unit: kg/head/year

| Feeding method | Cow | Non-dairy cow | Buffalo | Sheep | Goat | Pig | Horse | Donkey/mule | Camel |
| --- | --- | --- | --- | --- | --- | --- | --- | --- | --- |
| Large-scale feeding | 88.1 | 52.9 | 70.5 | 8.2 | 8.9 | 1 | 18 | 10 | 46 |
| Farmer scatter-feeding | 89.3 | 67.9 | 87.7 | 8.7 | 9.4 |  |  |  |  |
| Grazing feeding | 99.3 | 85.3 | — | 7.5 | 6.7 |  |  |  |  |

Table S4 Manure management CH_4_ emission factor

Unit: kg/head/year

| Region | Cow | Non-dairy cow | Buffalo | Sheep | Goat | Pig | poultry | Horse | Donkey/mule | Camel |
| --- | --- | --- | --- | --- | --- | --- | --- | --- | --- | --- |
| North China | 7.46 | 2.82 | — | 0.15 | 0.17 | 3.12 | 0.01 | 1.09 | 0.60 | 1.28 |
| Northeast China | 2.23 | 1.02 | — | 0.15 | 0.16 | 1.12 | 0.01 | 1.09 | 0.60 | 1.28 |
| East China | 8.33 | 3.31 | 5.55 | 0.26 | 0.28 | 5.08 | 0.02 | 1.64 | 0.90 | 1.92 |
| Central south China | 8.45 | 4.72 | 8.24 | 0.34 | 0.31 | 5.85 | 0.02 | 1.64 | 0.90 | 1.92 |
| Southwest China | 6.51 | 3.21 | 1.53 | 0.48 | 0.53 | 4.18 | 0.02 | 1.64 | 0.90 | 1.92 |
| Northwest China | 5.93 | 1.86 | — | 0.28 | 0.32 | 4.38 | 0.01 | 1.09 | 0.60 | 1.28 |

Table S5 Carbon emission parameter factors for various agricultural products.

|  | Economic coefficient | Moisture rate | Correction factor | Conversion factor |
| --- | --- | --- | --- | --- |
| Wheat | 0.35 | 0.125 | 0.07 | 0.5 |
| Paddy | 0.41 | 0.15 | 0.07 | 0.5 |
| Corn | 0.49 | 0.135 | 0.07 | 0.5 |
| Soy | 0.25 | 0.125 | 0.07 | 0.5 |
| Cotton | 0.35 | 0.083 | 0.07 | 0.5 |
| Oil | 0.39 | 0.09 | 0.07 | 0.5 |
| Vegetables | 0.7 | 0.65 | 0.07 | 0.5 |
